# *In silico* biomarker analysis of the adverse effects of perfluorooctane sulfonate (PFOS) exposure on the metabolic physiology of embryo-larval zebrafish

**DOI:** 10.3389/fsysb.2024.1367562

**Published:** 2024-03-27

**Authors:** Rayna M. Nolen, Lene H. Petersen, Karl Kaiser, Antonietta Quigg, David Hala

**Affiliations:** ^1^ Department of Marine Biology, Texas A&M University at Galveston, Galveston, TX, United States; ^2^ Department of Marine and Coastal Environmental Science, Texas A&M University at Galveston, Galveston, TX, United States; ^3^ Department of Oceanography, Texas A&M University, College Station, TX, United States; ^4^ Department of Ecology and Conservation Biology, Texas A&M University, College Station, TX, United States

**Keywords:** systems toxicology, stoichiometric model, flux balance analysis, biomarkers, carnitine shuttle

## Abstract

Perfluorooctane sulfonate (PFOS) is a ubiquitous pollutant in global aquatic ecosystems with increasing concern for its toxicity to aquatic wildlife through inadvertent exposures. To assess the likely adverse effects of PFOS exposure on aquatic wildlife inhabiting polluted ecosystems, there is a need to identify biomarkers of its exposure and toxicity. We used an integrated systems toxicological framework to identify physiologically relevant biomarkers of PFOS toxicity in fish. An *in silico* stoichiometric metabolism model of zebrafish (*Danio rerio*) was used to integrate available (published by other authors) metabolomics and transcriptomics datasets from *in vivo* toxicological studies with 5 days post fertilized embryo-larval life stage of zebrafish. The experimentally derived omics datasets were used as constraints to parameterize an *in silico* mathematical model of zebrafish metabolism. *In silico* simulations using flux balance analysis (FBA) and its extensions showed prominent effects of PFOS exposure on the carnitine shuttle and fatty acid oxidation. Further analysis of metabolites comprising the impacted metabolic reactions indicated carnitine to be the most highly represented cofactor metabolite. Flux simulations also showed a near dose-responsive increase in the pools for fatty acids and acyl-CoAs under PFOS exposure. Taken together, our integrative *in silico* results showed dyslipidemia effects under PFOS exposure and uniquely identified carnitine as a candidate metabolite biomarker. The verification of this prediction was sought in a subsequent *in vivo* environmental monitoring study by the authors which showed carnitine to be a modal biomarker of PFOS exposure in wild-caught fish and marine mammals sampled from the northern Gulf of Mexico. Therefore, we highlight the efficacy of FBA to study the properties of large-scale metabolic networks and to identify biomarkers of pollutant exposure in aquatic wildlife.

## 1 Introduction

The health of organisms living in stressed or deteriorating ecosystems is typically assessed using biomarkers that represent normal vs. perturbed physiological functions (Lopez-Barea, 1995; Dahlhoff, 2004; Sarkar et al., 2006; Kerambrun et al., 2011; Kroon et al., 2017). Biomarkers can include the measurement of apical or biochemical endpoints that are representative of organismal fitness, such as survival, growth, and fecundity (Villeneuve and Garcia-Reyero, 2011), or use high-throughput “omics” methods (transcriptomics, proteomics, metabolomics) to assess the entirety of an organism’s biological complexity (Iguchi et al., 2005; Brinke and Buchinger, 2017). The datasets generated from omics analyses allows the generalized analysis of effects on biological pathways using various *in silico* (i.e., bioinformatics or computational biology) approaches (Ankley et al., 2010; Krewski et al., 2010; Moffat et al., 2015). However, an integrated physiological perspective is difficult to discern using conventional *in silico* pathways-based approaches as omics changes are mainly assigned to biological functions based upon ontological relationship (Kienhuis et al., 2011; Marmon et al., 2021).

At present, we lack a comprehensive framework for assessing the consequences of perturbations to specific biological pathways on organismal metabolic physiology (and likely fitness) (Ashburner et al., 2000; Hardy et al., 2012; Mi et al., 2013; Wolffe et al., 2020). Exceptions include when dose-response changes in select gene expressions relate to progressively worsening physiological outcomes (Waterman et al., 2010; Haggard et al., 2016). Such as is seen for exposure to hepatic toxicants and the outcome of tumor development (Waterman et al., 2010; Haggard et al., 2016), or exposures to endocrine disrupting chemicals and increasing physiological dysfunction (Sharpe, 2006; Bergman et al., 2012; La Merrill et al., 2020). Therefore, there is a need to understand how the perturbation of select (or ensembles of) biochemical pathways (or metabolic sub-systems), affects large-scale biological networks, in turn impacting physiological functions. This challenge necessitates the use of *in silico* models and methods that can enable the qualitative or quantitative determination of the metabolites or metabolic fluxes (i.e., catalytic capabilities of enzymes in metabolic pathways) that are required to maintain the overall adaptive potential of an organism’s physiology under changing environmental conditions. Given the multi-level (genome to phenome) and multi-variate (transcriptome, proteome, metabolome) complexity of biological systems, the development and use of physiologically representative *in silico* models is an active area of research (Thiele et al., 2020; Gopalakrishnan et al., 2022).

In this manuscript we used an integrated systems toxicological framework to identify the physiologically relevant biomarkers of perfluorooctane sulfonate (PFOS) toxicity in fish. We used a novel *in silico* computational biology approach to integrate metabolomics and transcriptomics datasets from *in vivo* toxicological studies exposing zebrafish (*Danio rerio*) to PFOS, with an *in silico* stoichiometric model of whole-organism metabolism of zebrafish. PFOS was chosen for analysis as it is a near ubiquitous pollutant in global aquatic ecosystems (Muir and Miaz, 2021; Kurwadkar et al., 2022), with concern for its toxicity to aquatic wildlife (Beach et al., 2006; Ankley et al., 2021) and humans through inadvertent exposures (Hansen et al., 2016; Blake and Fenton, 2020; Panieri et al., 2022). Typically, human and wildlife exposure to PFOS (and related per- and polyfluoroalkyl substances, or PFAS) can cause wide-ranging metabolic and endocrine disruptive effects (Ankley et al., 2021; Fenton et al., 2021). Therefore, the elucidation of biomarkers that can represent impacted biological pathways and portend the onset of adverse physiological effects, is a priority area for further research (Lee et al., 2020; Ankley et al., 2021).

The *in silico* approach taken in this manuscript involved integrating metabolomics and transcriptomics (RNA sequencing) datasets from *in vivo* toxicological studies, generated by other authors, in which embryo-larval life-stages of zebrafish were exposed to PFOS (Ortiz-Villanueva et al., 2018; Martínez et al., 2019). Zebrafish were chosen as a model organism due to their common use as an *in vivo* model for aquatic wildlife and human health (Hutchinson et al., 2003; Tanguay, 2018; Breuer and Patten, 2020). Untargeted metabolomics (Ortiz-Villanueva et al., 2018) and transcriptomics datasets (Martínez et al., 2019) from 5 days post fertilized (dpf) embryo-larval zebrafish exposed to PFOS (from 2 – 5 dpf) were used for model parameterization. Specifically, the omics datasets from both studies comprised equivalent exposure concentrations of a solvent control (0.2% ^v^/_v_ dimethyl sulfoxide or DMSO), 0.06, 0.6, or 2 µM PFOS, and were generated by the same research laboratory (Ortiz-Villanueva et al., 2018; Martínez et al., 2019). These datasets were used to constrain or parameterize an *in silico* stoichiometric model of zebrafish metabolism (Wang et al., 2021).

The zebrafish metabolism model comprised a stoichiometric matrix which balanced the conversions of 8,344 metabolites in 12,909 interlinked metabolic reactions, and with 61% of these reactions controlled by the transcripts of 2,714 genes (Wang et al., 2021). Analysis methods from the constraints-based reconstruction and analysis (COBRA) metabolic modeling framework were used to parameterize the zebrafish model and simulate the impacts of PFOS exposure on the metabolic physiology of zebrafish (Savinell and Palsson, 1992; Schellenberger et al., 2011; Lewis et al., 2012). The COBRA framework has been successfully used to study the optimal metabolic functions of various prokaryotic and eukaryotic organisms (including multi-tissue human metabolic models) (Ibarra et al., 2002; Fong et al., 2005; Duarte et al., 2007; Mo et al., 2009; Bordbar et al., 2011; Yim et al., 2011; Swainston et al., 2013; Aurich et al., 2015; Blais et al., 2017; Opdam et al., 2017; Herrmann et al., 2019; Thiele et al., 2020). Therefore, the *in silico* approach taken in this study enabled assessment of the adverse effects of PFOS exposure and helped to identify likely biomarkers of its toxicity effects in exposed aquatic wildlife. In particular, a metabolite biomarker discovered using the *in silico* analyses was later confirmed to be representative of PFOS exposure in aquatic wildlife (fish and dolphins) sampled from the northern Gulf of Mexico, namely, Galveston Bay (TX) and Mobile Bay (AL) (Nolen et al., 2024).

## 2 Materials and methods

### 2.1 The zebrafish metabolic model

A zebrafish metabolic model constructed by Wang et al. (2021) was parameterized with *in vivo* experimental data and used for subsequent *in silico* simulations (overall approach is summarized in Supplementary Material S1). The model mathematically related the biochemical (i.e., enzyme catalyzed) conversions and transport of 8,344 metabolites through an interlinked network of 12,909 reactions, and with 61% of these reactions related to the transcripts of 2,714 genes (SSupplementary Material S2, Zebrafish Metabolic Model). The mathematical model of the metabolic network was as a m x n dimension stoichiometric matrix in which the enzyme catalyzed biochemical conversions of “ m ” metabolites in “ n ” reactions was represented using negative or positive coefficients to encode metabolite consumptions or productions respectively. Approximately 13% of the reactions in the model comprised “Exchange” reactions that represented the uptake and/or excretion of various metabolites into or out of the model. The min/max catalytic capabilities (or bounds) for all reactions in the metabolic model were initially constrained with arbitrary or experimentally derived data of metabolite availabilities (Supplementary Material S2, Zebrafish Metabolic Model). Taken together, the stoichiometric matrix and the min/max reaction bounds defined the feasible domain of allowable and attainable metabolic fluxes that can convert substrates to products (Varma and Palsson, 1994). The imposition of experimentally derived constraints to these min/max (or lower/upper) bounds enabled the generation of condition-specific models that represented a given phenotype or experimental condition (as described in Sections 2.2–2.5).

### 2.2 Determination of *in vivo* biomass composition of 5 dpf zebrafish for *in silico* base-model parameterization

To create a base-model parameterized to the metabolic physiology of 5 dpf embryo-larval zebrafish, a combination of existing data (i.e., published by other authors) and new data generated by us for this study was used. For example, the proportional contributions of major biomass components required to generate per Gram dry weight (g DW) of 5 dpf embryo-larval zebrafish was estimated using methods detailed in Feist et al. (2007). Data on 5 dpf zebrafish protein and lipid fractions were obtained from Hachicho et al. (2015), trace ions from Kaushik et al. (2011), and the proportion of DNA was estimated from the genome size (in base pairs) as presented in the zebrafish genome assembly GRCz11 (http://uswest.ensembl.org/Danio_rerio/Info/Annotation) and converted to unit mass composition (Gregory et al., 2007). The organic acids pool was calculated as the remainder proportional fraction (all calculations are shown in Supplementary Material S2, Biomass Composition).

The specific amino acids, organic acids, and fatty acids comprising the protein, organic acid pool, and lipid fractions respectively for 5 dpf zebrafish were quantified in this study using untargeted metabolomics. Briefly, 100 newly fertilized wild-type (AB line) zebrafish embryos were purchased from the Zebrafish International Resource Center (ZIRC) and maintained until 5 dpf in E2 embryo medium (Westerfield, 2000). At 5 dpf, 50 cold-stunned embryo-larval zebrafish were pooled into two separate replicates, freeze-dried overnight (Labconco FreeZone 6 plus), with metabolites extracted into crushed dry ice/acetone using a pestle and mortar. The mixed homogenates were pelleted at 2,000 rcf for 10 min, with the acetone supernatants filtered at 12,000 rcf for 1 h (at 4°C) through Amicon Ultra-0.5 centrifugal filters (Millipore-Sigma). The resulting filtrates were analyzed using mass spectrometry by the Integrated Metabolomics Analysis Core (IMAC) at Texas A&M University.

Untargeted liquid chromatography high resolution accurate mass spectrometry (LC-HRAM) analysis was performed using a Q Exactive Plus orbitrap mass spectrometer (ThermoFisher, Waltham, MA) coupled to a binary pump HPLC (UltiMate 3,000, ThermoFisher). Full MS spectra was obtained at 70,000 resolution (200 ^m^/_z_) with a scan range of 50–750 ^m^/_z_. Full MS followed by data dependent (dd) MS2 scans were obtained at 35,000 resolution (MS1) and 17,500 resolution (MS2), with a 1.5 ^m^/_z_ isolation window and a stepped normalized collision energy (NCE of 20, 40, 60). Samples were maintained at 4°C before injection. The injection volume was 10 µL. Chromatographic separation was achieved on a Synergi Fusion 4 μm, 150 mm × 2 mm reverse phase column (Phenomenex, Torrance, CA) maintained at 30°C using a solvent gradient method. Sample acquisition was performed using Xcalibur (ThermoFisher). Data analysis was performed with Compound Discoverer 2.1 (ThermoFisher). The averaged area under curve (AUC) counts for the various biomass components of amino acids (protein fraction), intermediate metabolites (organic acids fraction), and fatty acids (lipid fraction); along with amounts of trace ions and nucleic acids, were converted to µmol gDW^-1^ zebrafish. The molar conversion to biomass constituents was based upon the protocols of Feist et al. (2007) and are detailed in Supplementary Material S2, Biomass Composition.

### 2.3 Determination of *in vivo* growth and metabolic rate of 5 dpf zebrafish for *in silico* base-model parameterization

Zebrafish growth rate was calculated by considering the change in total body length (mm) over time from ∼2 to 21 dpf, using data from https://zfin.org/zf_info/zfbook/stages/and Kimmel et al. (1995). The relationship between total body length and time was approximately linearized by plotting the cumulative change in total body length (mm) vs. the cumulative length increment per unit time (mm hr^-1^) (Supplementary Material S2, Growth and Metabolic Rate) (Hopkins, 1992). An oxygen consumption rate (OCR) for 5 dpf zebrafish was also calculated using the allometric relationship between routine (or resting) metabolic rate of zebrafish vs. mass (Rombough and Drader, 2009). And a cost of growth associated ATP maintenance (or COG) was calculated from the correlation between the OCR vs. relative growth rate (RGR) for zebrafish developmental life-stages spanning from 4 to 30 dpf. The RGR and COG were calculated using methods described in Conceição et al. (1998) and are detailed in Supplementary Material S2, Growth and Metabolic Rate. All physiological parameters were used to constrain the *in silico* stoichiometric model to generate a base-model that was representative of the metabolic physiology of 5 dpf zebrafish.

### 2.4 Use of omics data from *in vivo* PFOS exposed 5 dpf zebrafish for *in silico* model parameterization

To convert the 5 dpf zebrafish base-model to condition-specific model’s representative of PFOS exposure, metabolomics and transcriptomics datasets from Martínez et al. (2019) and Ortiz-Villanueva et al. (2018) exposing 5 dpf embryo-larval zebrafish to a solvent control (0.2% ^v^/_v_ DMSO), 0.06, 0.6, or 2 µM PFOS, were obtained and prepared for base-model parameterization. Specifically for the metabolomics datasets, the auto-scaled relative abundances for the up- or downregulated metabolite levels (as reported in Supplementary Material of Ortiz-Villanueva et al. (2018)) were used to scale the AUC’s for the matched metabolites measured in the wild-type zebrafish (as described in Section 2.2) (Supplementary Material S2, PFOS Omics Data). The GSE125072 transcriptomics (RNA-sequencing) datasets of 5 dpf zebrafish exposed to a solvent control (0.2% ^v^/_v_ DMSO), 0.06, 0.6, or 2 µM PFOS were downloaded from the NCBIs GEO website (GSE125072 dataset as FASTQ files) using the NCBIs sequence read archive (SRA) Toolkit as initialized in a Cygwin-64 terminal. The resulting FASTQ files were processed using the OmicsBox bioinformatics toolbox v1.2 using established algorithms (BioBam, Valencia, Spain). For example, sequence alignment was performed using the STAR (v.2.7.9) aligner (Dobin et al., 2013), and using the zebrafish reference genome GRCz11. Gene expression quantification as a transcript count table was performed using the HTSeq package (Anders et al., 2015). Subsequently, all counts were log_2_ (n + 1) transformed prior to binarization using the BiTrinA toolbox v1.3 (Mundus et al., 2022) in RStudio (v4.2.0), and using the Binarization Across multiple SCales or BASC (A) algorithm (transcriptomics and binarized data is shown in Supplementary Material S2, PFOS Omics Data).

### 2.5 The generation of PFOS exposed condition-specific zebrafish metabolic models

The zebrafish base-model was converted to condition-specific metabolic models representative of the various PFOS exposure concentrations (i.e., 0.2% ^v^/_v_ DMSO (solvent control), 0.06, 0.6, 2 µM PFOS) using the MetaboTools protocols (Aurich et al., 2015; Aurich et al., 2016), and as enabled in the COBRA toolbox (Schellenberger et al., 2011). All protocols were initialized in MATLAB (vR 2021b). The key parameterization steps are summarized as follows: First, the previously developed base-model was transformed to condition-specific model’s representative of PFOS exposure by constraining metabolite availabilities to generate various (or transformed) models reflective of the solvent control (0.2% ^v^/_v_ DMSO), 0.06, 0.6, or 2 µM PFOS exposure concentrations (n = 2 per treatment group) (Supplementary Material S2, PFOS Omics Data). Second, binarized transcriptomics datasets (described in Section 2.4) was used to parameterize the viable min/max bounds for reactions associated with the expressed metabolic enzyme genes only (and with nulled min/max bounds for the remainder of reactions). Third, imposition of the previously stated constraints led to the generation of three condition-specific metabolic models that were representative of PFOS exposures, namely, a Low exposure model (solvent control vs. 0.06 µM PFOS), Medium model (solvent control vs. 0.6 µM PFOS), and High model (solvent control vs. 2 μM PFOS) (Supplementary Material S2, PFOS Omics Data). While replicate datasets were used for model parameterization, single PFOS condition-specific models were generated using the MetaboTools protocols. Once constructed, the functional properties of each condition-specific metabolic model were studied in turn (all models are available in SBML format at https://www.ebi.ac.uk/biomodels/MODEL2403010004).

### 2.6 *In silico* biomarker discovery using condition-specific PFOS exposure metabolic models

The structural and functional properties of each condition-specific metabolic model was studied using the COBRA toolbox (v2.0) as initialized in MATLAB (v2021b) (Schellenberger et al., 2011). For each model, the full range of min/max flux constraints required for the optimal production of ATP was determined using the flux variability analysis (FVA) computation (Mahadevan and Schilling, 2003). The production of ATP by each model was selected as a proxy of metabolic performance due to its positive correspondence with *in vivo* metabolic rate and organismal fitness (Salin et al., 2015) (Supplementary Material S2, Flux Analysis). The extent of intersection or overlap of the computed min/max flux ranges were compared across the three condition-specific metabolic models using a Jaccard index calculation. This analysis allowed identification of impacted reactions and associated metabolic sub-systems under PFOS exposure. Further analysis was performed to determine the extent of metabolite participation in the impacted reactions. Therefore, providing insights into metabolite connectivity in the condition-specific models and helping to identify candidate metabolite biomarkers that were diagnostic of PFOS exposure. Finally, flux balance analysis (FBA) was used to compute the optimal flux through select core metabolic reactions that produced key intermediate organic acids or lipids (Feist and Palsson, 2010; Orth et al., 2010). Optimal fluxes were calculated subject to limiting carbon substrate availability to per mol glucose or palmitolate (or palmitoleic acid), and ensuring unlimited O_2_, H_2_O, and CO_2_ availability to each condition-specific model.

### 2.7 Statistical analysis

Statistical analysis was performed using the Python programing language (v3.9.5), with associated data handling (pandas) and statistical (scipy, scikit) libraries. The normal distribution of data was tested using the Shapiro-Wilk test, with homogeneity of variance tested using the Levene test. In this manuscript, a non-parametric Spearman’s rank correlation only was tested (with α = 0.05). All graphs were plotted using python’s matplotlib visualization library.

## 3 Results

### 3.1 The metabolic physiology of the 5 dpf embryo-larval zebrafish base-model

The proportional contributions of major biomass components comprised: organic acid (0.47), protein (0.40), lipid (0.05), trace ion (0.05), and DNA (0.03) fractions (Supplementary Material S2, Biomass Composition). The inverse slope of the relationship between the cumulative change in total body length vs. the cumulative body length increment (Spearman Rank rho = −0.84, *p*-value = 0.002) yielded a growth rate of 0.011 h^-1^ (Supplementary Material S2, Growth and Metabolic Rate). An OCR of 52 µmol O_2_ gDW^-1^ hr^-1^ and COG of 209 µmol ATP gDW^-1^ hr^-1^ was calculated for 5 dpf embryo-larval zebrafish (Supplementary Material S2, Growth and Metabolic Rate). The OCR was calculated using the allometric relationship between the resting metabolic rate (nmol O_2_/hr) and mass (mg) of larval zebrafish as calculated by Rombough and Drader (2009). Our computed OCR value (using the allometric relationship) was similar to the experimentally measured OCR of ∼30–40 µmol O_2_ g wet weight^-1^ hr^-1^ for 5 dpf embryo-larval zebrafish reported in Bagatto et al. (2001). All experimentally derived physiological parameters were used as constraints to generate the *in silico* metabolic base-model for 5 dpf zebrafish. For example, the *in vivo* growth rate was used to constrain the lower bound of the *in silico* biomass generating reaction (MAR00021), the OCR was used to constrain the O_2_ uptake exchange reaction (MAR09048), and the COG was used to constrain the ATP maintenance (or renewal) reaction (MAR06916) (Supplementary Material S2, Zebrafish Metabolic Model).

### 3.2 Comparisons amongst condition-specific PFOS exposed 5 dpf zebrafish metabolic models

**FIGURE 1:**
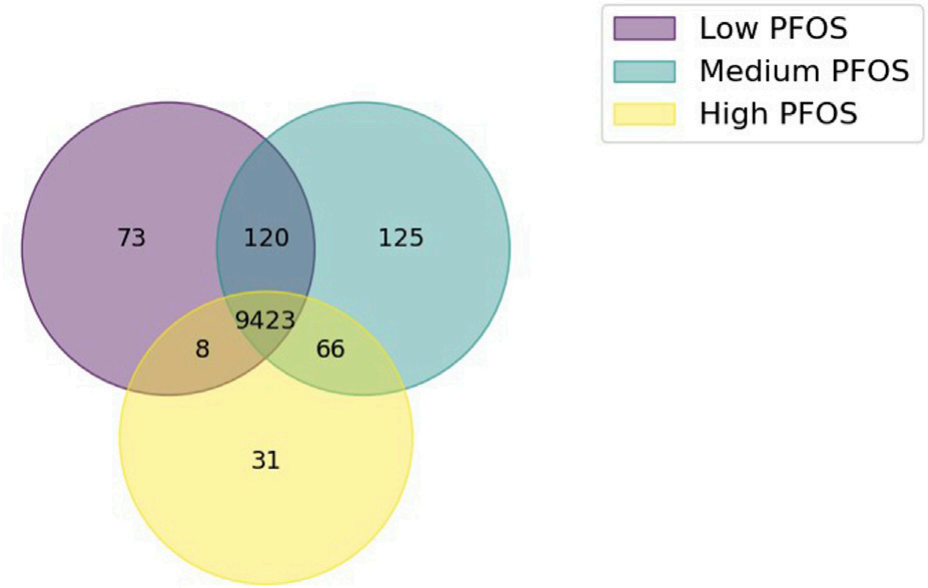
Venn diagram showing the extent of shared or unique metabolic reactions in the three condition-specific models generated using *in vivo* omics data from PFOS exposed embryo-larval zebrafish. The condition-specific metabolic models comprised Low PFOS (solvent control vs. 0.06 µM PFOS), Medium PFOS (solvent control vs. 0.6 µM PFOS), and High PFOS (solvent control vs. 2 μM PFOS).

The analysis of the union of reactions across the three, condition-specific metabolic models yielded a shared pool of 9,846 reactions, which constituted 76% of all reactions in the zebrafish base-model. A Venn diagram of shared or unique reactions amongst the condition-specific models revealed that 96% of metabolic reactions to be shared amongst the three models (Figure 1). Relative to the union of all reactions, the set of unique reactions was relatively small in each of the Low (0.7%), Medium (1.3%), or High (0.3%) PFOS exposed metabolic models. And while approximately twice as many reactions were shared between the Low and Medium models (120) vs. the Medium and High models (66), they only comprised 1.2% and 0.7% of all shared reactions (Figure 1). Therefore, given the relatively minimal distinctions in reactions between the three condition-specific models, comparisons of the simulated metabolic fluxes were sought to identify the likely effects of PFOS exposure on the metabolic physiology of zebrafish.

### 3.3 PFOS effects on the *in silico* metabolic physiology of zebrafish

**FIGURE 2:**
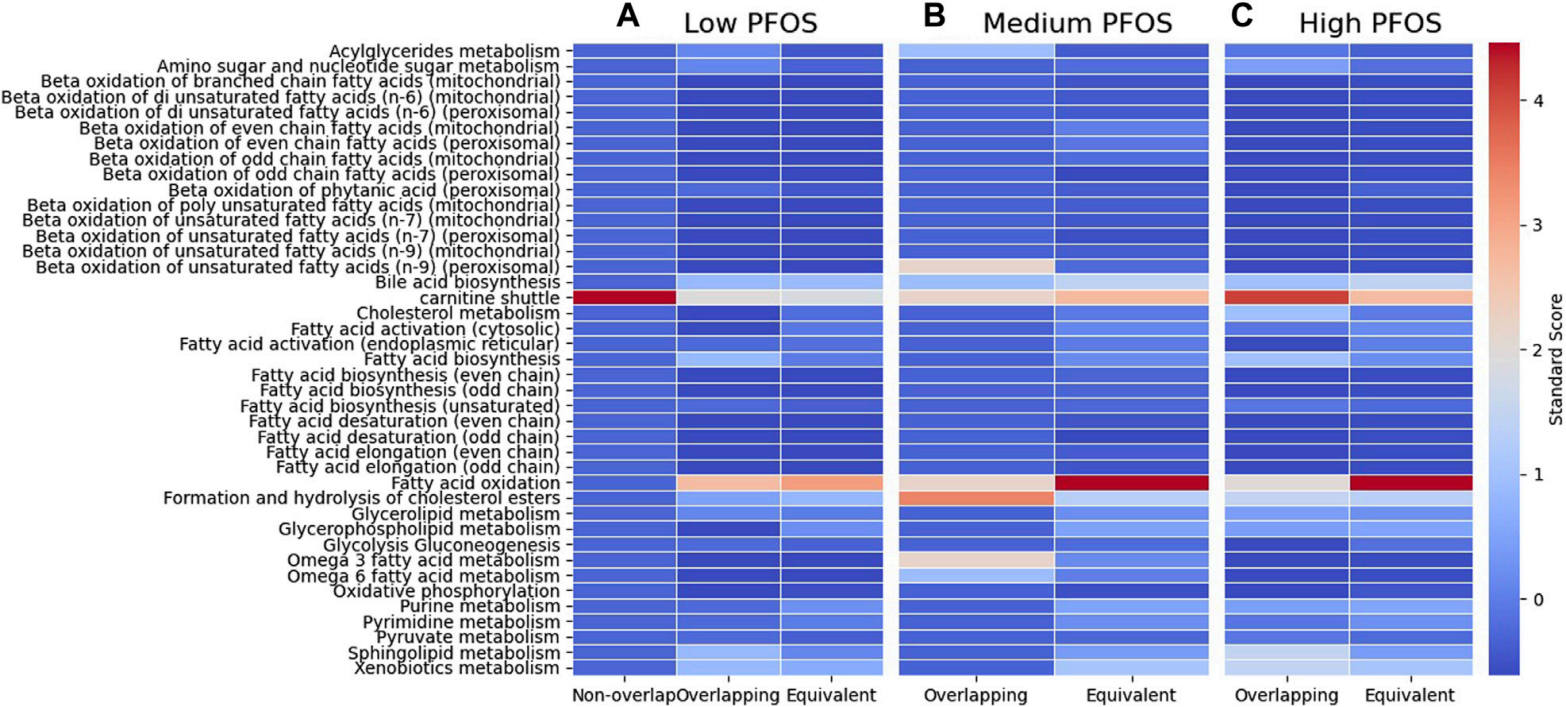
Comparisons of min/max flux overlap across various metabolic sub-systems as represented in the zebrafish metabolic model. The heat map shows the extent to which (darker colors) a metabolic sub-system is represented in disjoint or Non-overlapping min/max flux ranges, Overlapping, or Equivalent (with equal min/max flux ranges). **(A)** Low PFOS = solvent control vs. 0.06 μM PFOS model, **(B)** Medium PFOS = solvent control vs. 0.6 μM PFOS model, and **(C)** High PFOS = solvent control vs. 2 μM PFOS model.

The comparisons of min/max flux values for the reactions comprising the major metabolic sub-systems revealed a prominent effect on the carnitine shuttle (Figure 2). The Low PFOS exposure model showed the carnitine shuttle to be uniquely impacted as its min/max fluxes were non-overlapping with those of all other reactions in the Low condition-specific model. The carnitine shuttle sub-system also appeared to be consistently affected (or represented) across all metabolic models. Given that carnitine availability is essential for the regulation of fatty acid oxidation (Ramsay and Zammit, 2004; Longo et al., 2016), the other major metabolic sub-system to also be concomitantly impacted across all three condition-specific metabolic models included fatty acid oxidation (Figure 2).

**FIGURE 3:**
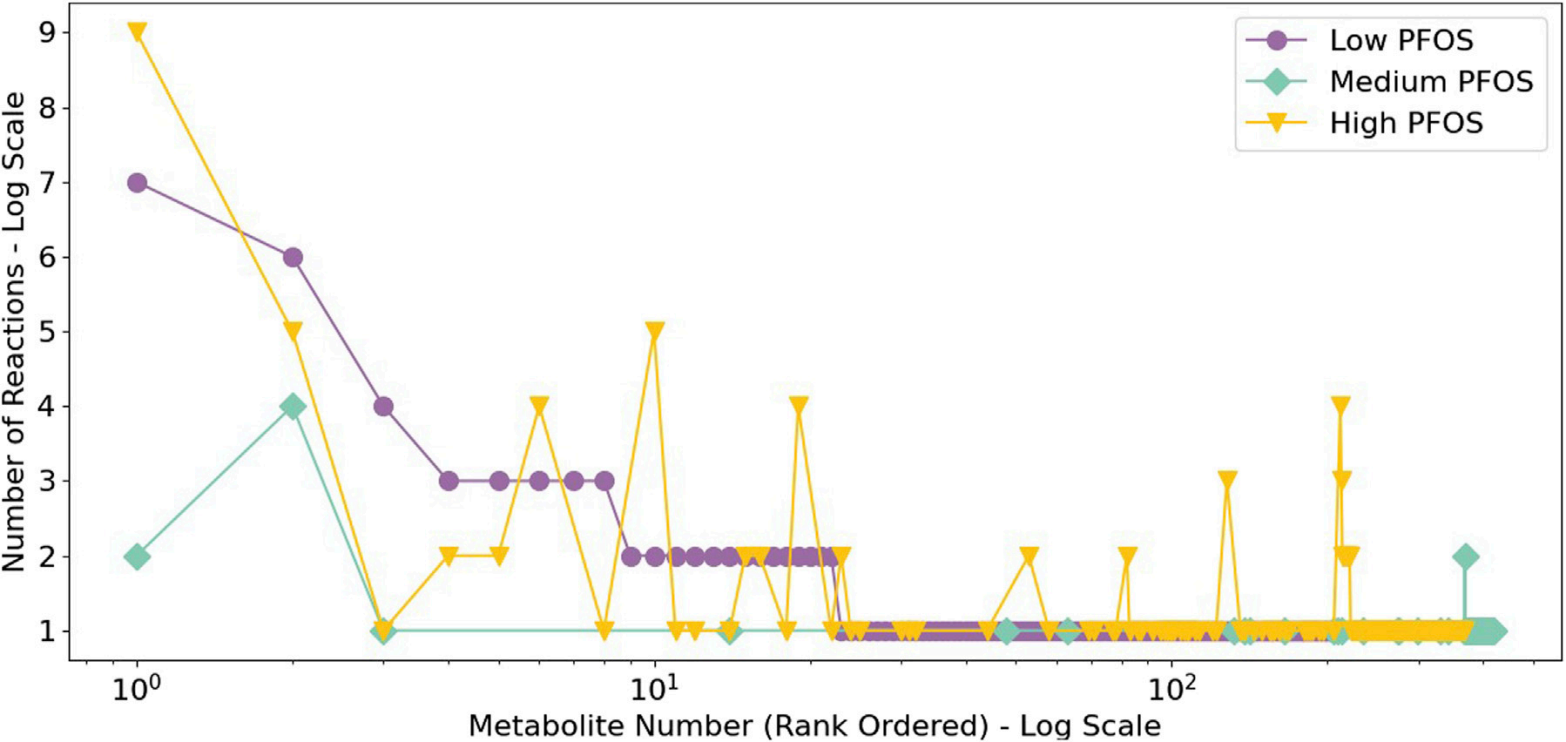
Rank ordered metabolites listed based upon the extent of their connectivity or participation in the metabolic reactions with disjoint or Non-overlapping and Overlapping min/max flux ranges. This analysis identified carnitine to be highly represented in the Low and High PFOS metabolic models. (Low PFOS = solvent control vs. 0.06 µM PFOS model, Medium PFOS = solvent control vs. 0.6 µM PFOS model, and High PFOS = solvent control vs. 2 μM PFOS model.).

The analysis of metabolite connectivity or participation in the impacted metabolic reactions (i.e., non-overlapping and overlapping flux categories) revealed highly represented or conserved biomarkers that were diagnostic of PFOS exposure (Figure 3). An evaluation of highly represented metabolites in the Low PFOS exposed model included: carnitine (or L-carnitine) > cholesterol > malonyl-CoA; for the Medium PFOS model included only cholesterol; and for the High PFOS model included: carnitine > cholesterol > acetyl-CoA > SM-pool (sphingolipid metabolism) > PC-LD pool (glycerophospholipid metabolism) > GSH (glutathione). The prominent representation of carnitine in the Low and High PFOS models implicates impacts of PFOS exposure on long-chain fatty acyl-CoA transmembrane import into the mitochondria and subsequent β-oxidation (Gooding et al., 2004; Longo et al., 2016). The participation of additional intermediate metabolites that are on the nexus of lipid and energy metabolisms (i.e., acetyl-CoA, malonyl-CoA, cholesterol), strongly implicated adverse effects on dyslipidemia and organismal metabolic physiology (Erinc et al., 2021; Fenton et al., 2021).

**FIGURE 4:**
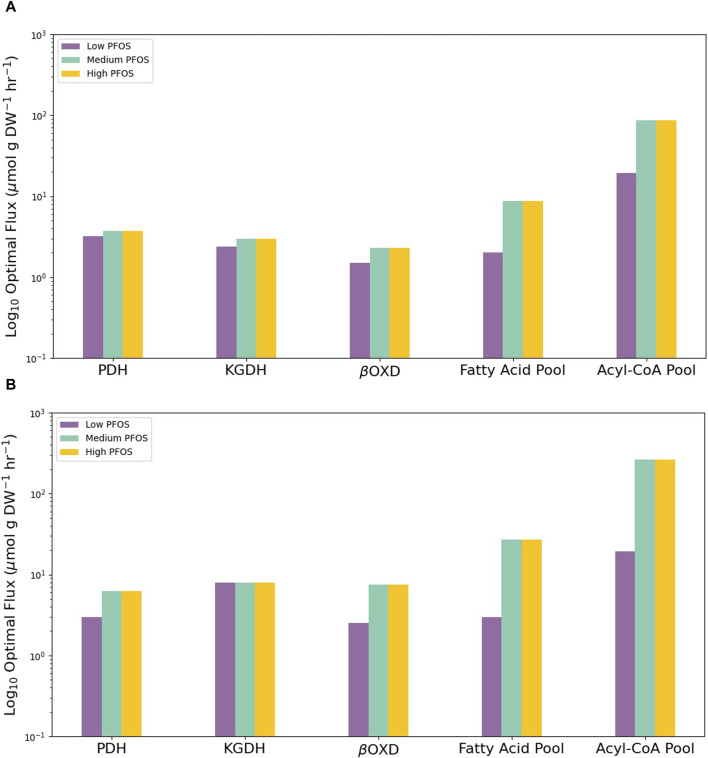
Graphs showing the contrasting abilities of the three condition-specific PFOS metabolic models to drive flux through key metabolic reactions or produce key biomass precursors under limited carbon availability of **(A)** 1 mol glucose or **(B)** 1 mol palmitolate to produce ATP subject to the availability of various organic carbon substrates. Low PFOS = solvent control vs. 0.06 µM PFOS model, Medium PFOS = solvent control vs. 0.6 µM PFOS model, and High PFOS = solvent control vs. 2 μM PFOS model. PDH = pyruvate dehydrogenase; KGDH = α-ketoglutarate dehydrogenase, βOXD = mitochondrial β-oxidation reaction of 3-ketoacyl-CoA thiolase.

Finally, the metabolic capability of each condition-specific model to produce key biomass precursors was tested using FBA. Specifically, limiting carbon substrate availability to per mol glucose or palmitolate showed the Medium and High PFOS metabolic models to exhibit overall higher fluxes for lipid metabolism vs. for those for organic acids (pyruvate dehydrogenase or PDH; and α-ketoglutarate dehydrogenase or KGDH) (Figure 4). For example, fatty acid oxidation via the mitochondrial β-oxidation reaction of 3-ketoacyl-CoA thiolase (βOXD), and the biomass generating reaction pools for fatty acids and acyl-CoAs was approximately an order of magnitude higher in the Medium and High models vs. the Low model (Figure 4). These flux simulations indicate increased lipid metabolism under elevated PFOS exposure.

## 4 Discussion

### 4.1 *In silico* predictions of PFOS exposure biomarkers

In this manuscript, the analysis of condition-specific zebrafish metabolic models revealed the carnitine shuttle and fatty acid oxidation to be consistently modulated under PFOS exposure (Figure 2; Figure 3). This is not surprising as the two sub-systems are functionally interrelated. The carnitine shuttle involves the use of carnitine as a cofactor to transport acyl moieties of fatty acyl-CoAs across the mitochondrial outer and inner membranes. Carnitine is a hydrophilic quaternary amine endogenously produced from protein degradation (Longo et al., 2016; Li and Zhao, 2021). In the carnitine shuttle, the acyl moiety of acyl-CoAs is transferred from CoA to carnitine (enabled by carnitine palmitoyl transferase I or CPT-I) on the outer mitochondrial membrane. Acyl-carnitine transfer across the inner mitochondrial membrane culminates in its reconversion to acyl-CoA by carnitine palmitoyl transferase II (CPT-II), which is found on the inner (matrix facing) face of the inner mitochondrial membrane, whereas the enzyme carnitine acyl-carnitine translocase replenishes the replaced carnitine by shuttling it back across the mitochondrial membranes to the cytosol, thus renewing the cytosolic carnitine pool and preventing its depletion (Bartlett and Eaton, 2004; Houten and Wanders, 2010).

Modulation of the carnitine shuttle and fatty acid oxidation metabolic sub-systems strongly indicates PPARα induction. PPARα serves as a sensor of free fatty acid levels and is activated by the presence of a variety of saturated and unsaturated fatty acids (Berger and Moller, 2002). Once activated this receptor acts as a transcriptional master-regulator by up-regulating genes required for activating fatty acids to fatty acyl-CoAs (fatty acyl-CoA synthetase), and those involved in fatty acid transport into the cell (fatty acid transport proteins or FATPs), along with mitochondrial and peroxisomal β-oxidation genes that metabolize fatty acyl-CoAs to acetyl-CoA (Berger and Moller, 2002; Rakhshandehroo et al., 2010). As a result, PFOS mediated activation of PPARα is expected to be concomitant with elevated free fatty acid levels and fatty acyl-CoAs. In support of this expectation, carbon-restricted *in silico* FBA simulations using per mol glucose or palmitolate showed elevated metabolic flux (by almost an order of magnitude) through the fatty acid and fatty acyl-CoA pools in the Mid and High condition-specific PFOS metabolic models, relative to the Low PFOS model (Figure 4).

Taken together, our *in silico* results indicate dyslipidemia effects under PFOS exposure. The candidate metabolite biomarkers of PFOS exposure include carnitine, free fatty acids, or fatty acyl-CoAs. Whereas PPARα and its downstream target genes, particularly those involved with fatty acyl-CoA mobilization or breakdown in fatty acid oxidation may provide appropriate biomarkers for transcriptomics analyses. These biomarkers agree well with the experimental observations of Ortiz-Villanueva et al. (2018) and Martínez et al. (2019) from whose *in vivo* exposure studies with embryo-larval zebrafish, the metabolomics and transcriptomics datasets used for model parameterization were derived. While metabolomics analyses indicated effects on lipid metabolism (elevated glycerophospholipid, unsaturated fatty acids) (Ortiz-Villanueva et al., 2018), transcriptomics analyses indicated the induction of lipid transport and metabolism (Martínez et al., 2019). Of specific relevance to the *in silico* prediction of effects on carnitine (as co-factor) and the carnitine shuttle made here, a human epidemiological study by Hu et al. (2019) showed PFOS body-burdens (in maternal perinatal serum samples) to be uniquely associated with the over-representation of carnitine and activation of the carnitine shuttle (as determined from metabolomics analyses). These findings confirmed PFOS toxicity on lipid regulation and fatty acid metabolism (Hu et al., 2019). Interestingly, the serum concentrations of PFOS shown to illicit effects on the carnitine shuttle (≤200 ng mL^-1^ or ≤0.4 µm PFOS) (Hu et al., 2019), were within the *in vivo* toxicological concentrations used for *in silico* model parameterization in this study (i.e., 0.06, 0.6, 2 µM PFOS) (Ortiz-Villanueva et al., 2018; Martínez et al., 2019). Indicating a concentration-dependent conservation of adverse effects.

The relevance of any biomarker identified from laboratory-based toxicological assessments with model organisms (such as zebrafish), as being predictive of effects in unrelated aquatic wildlife species is likely to depend on the extent of genome or proteome sequence similarity (Lalone et al., 2013; LaLone et al., 2023). Interspecies differences in receptor sensitivity to a pollutant ligand has been clearly shown to relate to differences in the inducibility of metabolic systems and related toxicity (Rusyn et al., 2006; Feige et al., 2010). However, if there is conservation of response for effects on a particular metabolic sub-system, then evaluation of effects on key cofactor metabolites may provide a more generalized approach towards identifying biomarkers. Carnitine presents itself as one such biomarker for studying effects on fatty acid oxidation as it is indispensable for the mitochondrial import of fatty acyl-CoAs (Longo et al., 2016).

In environmental monitoring studies performed by the authors of this manuscript, hepatic carnitine levels were shown to positively correlate with PFOS body-burdens in fish and marine mammals (stranded dolphins) sampled from the northern Gulf of Mexico (namely, from Galveston Bay (TX) and Mobile Bay (AL)) (Nolen et al., 2024). Therefore, the *in silico* predictions presented in this manuscript and the subsequent *in vivo* experimental verification reported in Nolen et al. (2024), indicate that the dyslipidemia effects of PFOS exposure likely mediate through disruption of the carnitine shuttle and modulation of carnitine levels as cofactor. Furthermore, the apparent agreement between *in silico* and *in vivo* results highlights the efficacy of using FBA to study the physiologically representative properties of large-scale metabolic networks.

An additional cofactor such as Coenzyme A (CoA) may also be a candidate to explore as a biomarker for PFOS effects. CoA is also an essential cofactor responsible for activating carboxylic acid moieties on various intermediate metabolites (including fatty acids) to CoA-thioesters (or acyl-CoAs). In turn, acyl-CoAs participate in key oxidative and biosynthetic reactions (such as fatty acid β-oxidation and the TCA cycle) (Brass, 1994). Therefore, adjustments of free CoA/acyl-CoA ratios contribute to major redirections of carbon flux in healthy vs. stressed metabolic states, although such effects are likely to be inducible under very high levels of PFOS exposure, as appears to be the case under di (2-ethylhexyl) phthalate (DEHP) exposure (Sakurai et al., 1978; Hala et al., 2021).

### 4.2 Relevance of *in silico* predictions within context of reported *in vivo* PFOS toxicity effects

To frame the *in silico* predicted effects determined in this manuscript with the broader base of knowledge on the *in vivo* toxicity effects of PFOS, we find that exposure to PFOS impacts a wide array of biomarkers, spanning from metabolic to endocrine systems. Such effects are explainable by its endocrine disruptive properties (Jensen and Leffers, 2008; Pípal et al., 2022). While endocrine disrupting chemicals (or EDCs) typically affect hormone signaling (Bergman et al., 2012; La Merrill et al., 2020), a broader suite of effects including metabolic disruption can also be considered (Grün and Blumberg, 2009; Heindel et al., 2017). PFOS appears to disrupt hormone production and signaling (Du et al., 2013; Salgado-Freiría et al., 2018), and impacts lipid or carbohydrate metabolisms in exposed organisms (Bjork et al., 2011; Chung et al., 2022). A comprehensive review of PFOS effects on lipid metabolism by Fragki et al. (2021) summarizes PPARα activation to be responsible for dyslipidemia effects. PPARα plays a critical role in regulating fatty acid metabolism via peroxisomal and mitochondrial β-oxidation (Berger and Moller, 2002). However, detailed transcriptomics comparisons between primary human vs. rodent hepatocytes exposed to PFOS indicate strong and concomitant activations of a broader suite of nuclear hormone receptors between the two species. These receptors included the constitutive androstane receptor (CAR) and pregnane X receptor (PXR) (Bjork et al., 2011). CAR/PXR are responsive to xenobiotic (synthetic exogenous chemicals or pollutants) exposures as they induce biotransformation enzyme activities (Wang et al., 2012), and also play a role in energy homeostasis by inducing lipogenesis and inhibiting gluconeogenesis (Moreau et al., 2008). Taken together, the nexus of PPARα (albeit weakly activated in humans; Takacs and Abbott (2007)), CAR, and PXR activations under PFOS exposure implicate dysregulations of energy homeostasis (particularly dyslipidemia) and consequent widespread adverse physiological effects. Therefore, it is not surprising that dyslipidemia is a consistent metabolic effect reported in humans exposed to PFOS (Christensen et al., 2019; Sunderland et al., 2019).

Similarly, the study of PFOS effects on zebrafish supports dyslipidemia effects via elevated fatty acid β-oxidation and nuclear hormone receptor activations (including PXR) reported (Cheng et al., 2016). The analysis of specific effects on fatty acid profiles shows dose-responsive increases in the medium-chain and long-chain saturated fatty acids of lauric (12:0) and myristic (14:0) acid in embryo-larval zebrafish exposed to PFOS (Sant et al., 2021). Overall, while laboratory studies convincingly show effects of PFOS exposure of endocrine and metabolic disruption (Lee et al., 2020), less is known of aquatic wildlife effects in field-based studies (Ankley et al., 2021). This is not surprising given the myriad of confounding variables that can impact biomarker assessments in wild-caught animals (Houde et al., 2011). For example, Guillette et al. (2020) report elevated PFOS body burdens in the serum of Striped Bass (*Morone saxatilis*) from a PFAS polluted river (Cape Fear River, NC, USA), to positively correlate with biomarkers of innate immune activity and hepatic injury. However, these correlations are potentially confounded by life-stage differences in fish, pathogen exposures, and likely exposures to mixtures of pollutants (as the river basin was also recipient of industrial and municipal discharges) (Heintz and Haws, 2021).

The requirement of a toxicity biomarker to be representative of exposure and/or adverse effects (Sanajou and Şahin, 2021), places a biomarker’s response within close proximity of a toxicants interaction with a molecular or biological target (the so called molecular initiating event) (Ankley et al., 2010). Close correspondence of elevated PFAS exposure with the activation of a responsive target receptor has been shown for the positive correlation between levels of select perfluoroalkyl carboxylates (PFCAs) and PPARα gene expression in the kidney tissue of stranded cetaceans (dolphins and whales) (Kurtz et al., 2019). In addition, Kurtz et al. (2019) also showed a positive correlation of PPARα expression with a target gene, cytochrome P450 4A (cyp4A), which is responsible for fatty acid metabolism (Simpson, 1997), and therefore strongly supporting a biologically relevant biomarker response (Kurtz et al., 2019). The Kurtz et al. (2019) study demonstrates that consideration of a “systems” view, such as the activations of responsive or inducible biomarkers (i.e., PPARα and cyp4a) can provide compelling weight-of-evidence of exposure to a specific pollutant and likely mechanistically associated adverse effects.

In this manuscript, the *in silico* predictions of effects on the carnitine shuttle and fatty acid metabolism agree with the general consensus of PFOS’ effects on lipid metabolism. Furthermore, our subsequent verification of the positive correlation between *in vivo* hepatic carnitine levels and PFOS body-burdens in fish and dolphins sampled from the northern Gulf of Mexico (Nolen et al., 2024), lends to the verification and validation of the *in silico* analyses reported in this manuscript. While the zebrafish metabolism model only accounted for effects on the metabolic reaction network, it precluded direct assessment of effects on nuclear hormone receptors (such as PPARs, CAR, PXR, etc.). We may infer that the metabolic effects simulated in this manuscript are consequent of transcriptional regulatory changes that may include perturbation of various transcription factors. The inclusion of transcriptional regulatory signaling and effects onto the metabolism model constitutes a further avenue for model improvement and research.

### 4.3 Limits of assumptions of the *in silico* modeling approach

The zebrafish metabolic model represented the near entirety of organismal biochemistry (comprising 12,909 reactions) (Wang et al., 2021). As a result, the model did not represent a tissue-specific metabolic phenotype. This is not a limitation of the model as the omics datasets used for its parameterization represented whole-organism transcriptomics and metabolomics changes (Ortiz-Villanueva et al., 2018; Martínez et al., 2019). Furthermore, use of the MetaboTools application enabled the generation of a subset of condition-specific metabolic reactions that were representative of PFOS exposure. While the steady-state assumption of FBA presents a computationally efficient means to solve (or mathematically study the properties of) large scale metabolic models (Schilling et al., 1999; Orth et al., 2010), it precludes consideration of system dynamics (at least as parameterized in its current form). Such absent inclusion of system dynamics is considered a limitation of the FBA approach (Murphy et al., 2018). However, FBA and its extensions present an effective and unified mathematical framework with which to study the properties of large-scale metabolic networks. Specifically, the FBA framework can meaningfully integrate transcriptomics and metabolomics datasets as constraints to determine effects on metabolic phenotype (Lee et al., 2006; Lewis et al., 2012).

The high level of genomic orthology and biochemical conservation between zebrafish and humans ensures relevance to the predictions of adverse effects in humans (Howe et al., 2013; Breuer and Patten, 2020). And conservation of zebrafish endocrinology and biochemistry with other piscine species also makes it an effective model system to study other aquatic wildlife (Hutchinson et al., 2003; Busby et al., 2010). Overall, the results of our *in silico* analyses agree with the *in vivo* toxicological effects of PFOS exposure, with the cofactor carnitine uniquely identified as a likely conserved metabolic biomarker of PFOS effects on fatty acid oxidation or dyslipidemia.

Finally, consideration should also be given to the fact that there are over 3,000 structurally related PFAS homologs, which raises concerns for mixtures exposures and toxicity (Wang et al., 2017; Ankley et al., 2021). Despite such variety, only around two dozen or so PFAS are commonly detected in aquatic wildlife (and most of whom are aptly represented in the Environmental Protection Agency’s PFAS priority list (EPA, 2019)). And of these, PFOS is the most prominently detected (and at the highest concentrations) (extensively reviewed in Vendl et al. (2021)). Our own previous research has shown PFOS to be the most prominently detected homolog in aquatic biota (oysters and fish) from Galveston Bay (TX) (Nolen et al., 2022). Given that in humans and fish, PFAS exposures are commonly associated with dyslipidemia effects (Lee et al., 2020; Fenton et al., 2021), we may expect PFOS to be the most causal homolog for this etiology. Furthermore, studies assessing the mixtures toxicity of PFAS indicate additive or synergistic effects of exposure on dyslipidemia (Haug et al., 2023; Sadrabadi et al., 2024) and cell toxicity (Ojo et al., 2020; Addicks et al., 2023; Pierozan et al., 2023). Therefore, the prediction of carnitine as a likely biomarker of PFOS toxicity may also be a relatively modal or representative biomarker of general PFAS exposures.

## 5 Conclusion

An integrated *in silico* systems toxicological framework was used to identify the physiologically relevant biomarkers of PFOS toxicity in fish. FBA simulations revealed the carnitine shuttle and fatty acid oxidation to be consistently affected under PFOS exposure. Specifically, the co-factor carnitine was identified as being a diagnostic metabolite biomarker of PFOS exposure. These simulation predictions agree with *in vivo* toxicological studies using animal models (including fish) (Lee et al., 2020; Ankley et al., 2021), human epidemiological studies (Christensen et al., 2019; Hu et al., 2019; Sunderland et al., 2019), and our own environmental monitoring data that reports a positive correlation between hepatic carnitine levels and PFOS body-burdens in fish and dolphins sampled from the northern Gulf of Mexico (Nolen et al., 2024). A key finding of our study is that adverse effects on key cofactor metabolites may provide a generalized approach towards identifying causal biomarkers to assess pollutant exposure and toxicity.

## Data Availability

The datasets presented in this study can be found in online repositories. The names of the repository/repositories and accession number(s) can be found below: https://www.ncbi.nlm.nih.gov/, GSE125072.
