# Supplementary material for: In silico biomarker analysis of the adverse effects of perfluorooctane sulfonate (PFOS) exposure on the metabolic physiology of embryo-larval zebrafish
Source: Front Syst Biol. 2024 Mar 27;4:1367562. doi: 10.3389/fsysb.2024.1367562 (PMC12341969; doi:10.3389/fsysb.2024.1367562)
Supplement: Supplementary file 2 [file Table1.DOCX]

**Supplemental 1.**

The schematic below highlights key features of the experimental approach taken in this manuscript.


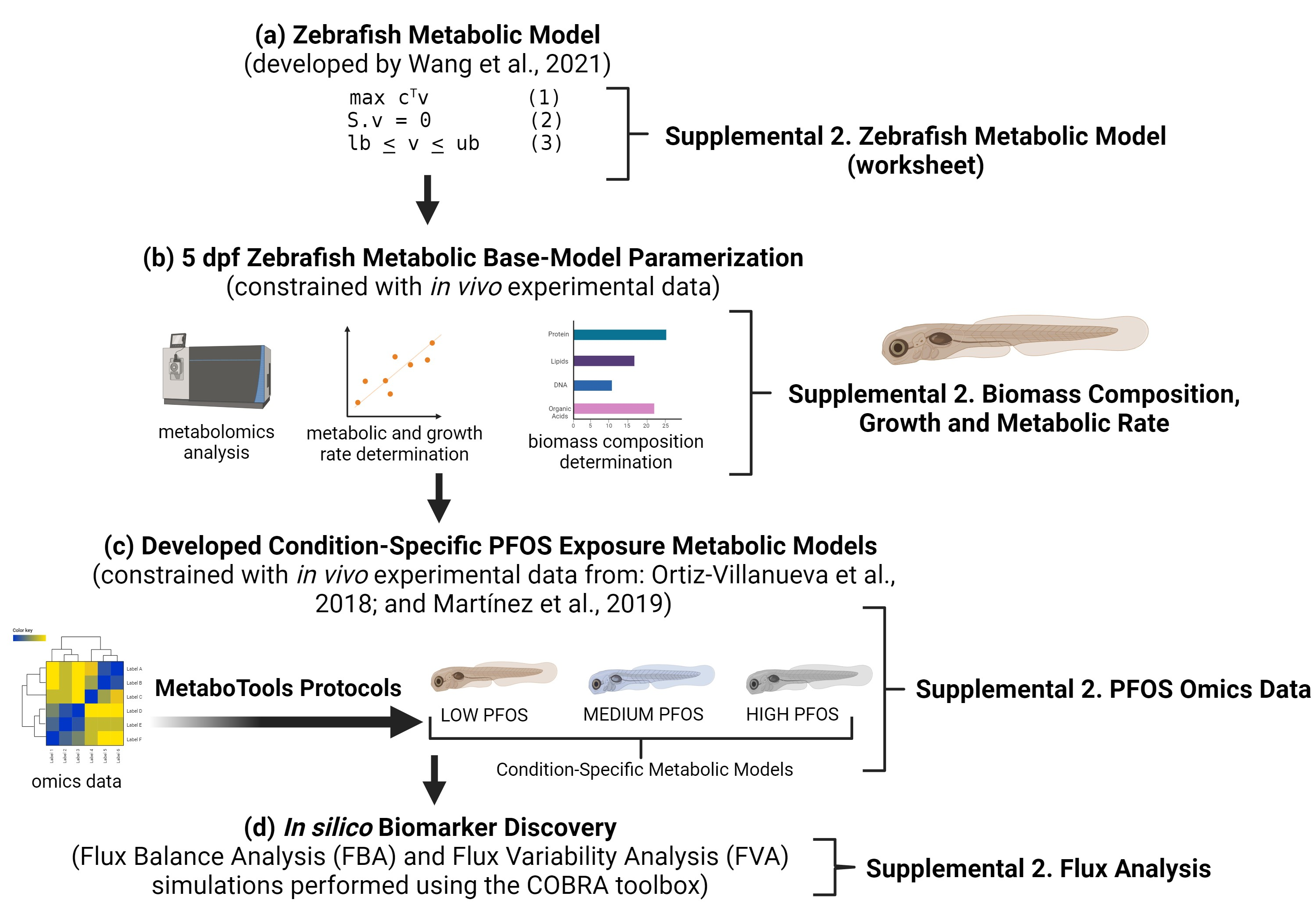


*First*, a stoichiometric model of zebrafish metabolism as developed by Wang et al., (2021) was used as a framework to study the effects of PFOS exposure on the metabolism of zebrafish (**Supplemental 2, Zebrafish Metabolic Model**). The stoichiometric matrix (or S-matrix) mathematically represented enzyme catalyzed reactions as a m x n dimension matrix. The S‑matrix described the consumption (negative integers) or production (positive integers) of metabolites (comprising the m x 1 row vector) in interrelated enzyme catalyzed reactions (represented along the 1 x n column vector). The solution to such a problem-description requires definition of a performance measure or ‘objective function’ whose value is selected for either maximization or minimization (i.e., max c^T^v in equation (1)). The objective function is a linear combination (as inner product) of a vector of transposed coefficients (c^T^) that indicates the system variable selected for optimization (i.e., a selected reaction). This objective is computed subject to the invariance of the reaction network at steady state (S.v = 0 in equation (2)), and application of linear inequality constraints, with minimum or lower bounds (lb_i_ < v_i_) and maximum or upper bounds (v_i_ < ub) imposed upon each reaction (v_i_) of the model (equation (3)). Taken together, equations (1) – (3) in the schematic are framed as a linear programming optimization problem and represented under the formalism of flux balance analysis or FBA (Orth et al., 2010).

*Second*, we used previously published data (by other authors), or new data generated by us to constrain the original Wang et al., (2021) zebrafish metabolic model to represent the metabolic physiology of 5 days post fertilized (dpf) embryo-larval zebrafish as a base-model. The details of how such data was generated are provided in the text of the manuscript with calculations shown in **Supplemental 2. Biomass Composition**, **Growth and Metabolic Rate**. The application of such data as constraints requires changing the min/max bounds for the uptake/excretion reactions (also called Exchange reactions) for selected (or representative) metabolites or reactions in the model.

*Third*, the base-model was further modified or re-constrained to reflect the metabolomics or transcriptomics changes quantified by other authors in embryo-larval zebrafish exposed to various PFOS concentrations, up to 5 dpf (as described in the text of the manuscript). Specifically, metabolomics data from Ortiz-Villanueva et al., (2018) and transcriptomics data (RNA-sequencing) from Martinez et al., (2019) was used for model parameterization (the re-constrained model parameters are detailed in **Supplemental 2. PFOS Omics Data**). Once constrained, the MetaboTools toolbox was used to transform the base-model to condition-specific metabolic models that were representative of the LOW, MEDIUM, and HIGH PFOS exposure groups (i.e., representative of 0.06, 0.6, or 2 µM PFOS). The multi-step implementation of MetaboTools command lines (as enabled in MATLAB) are described in Aurich et al., (2016). Once constructed Flux Balance Analysis (FBA) and its extensions, such as Flux Variability Analysis (FVA) was used to simulate properties of the various metabolic models (**Supplemental 2. Flux Analysis**).

**References**

Aurich, M.K., Fleming, R.M.T., Thiele, I., 2016. MetaboTools: A Comprehensive Toolbox for Analysis of Genome-Scale Metabolic Models. Front Physiol 7, 327-327.

Martínez, R., Navarro-Martín, L., Luccarelli, C., Codina, A.E., Raldúa, D., Barata, C., Tauler, R., Piña, B., 2019. Unravelling the mechanisms of PFOS toxicity by combining morphological and transcriptomic analyses in zebrafish embryos. Sci Total Environ 674, 462-471.

Orth, J.D., Thiele, I., Palsson, B.O., 2010. What is flux balance analysis? Nat Biotechnol 28, 245-248.

Ortiz-Villanueva, E., Jaumot, J., Martínez, R., Navarro-Martín, L., Piña, B., Tauler, R., 2018. Assessment of endocrine disruptors effects on zebrafish (Danio rerio) embryos by untargeted LC-HRMS metabolomic analysis. Sci Total Environ 635, 156-166.

Wang, H., Robinson, J.L., Kocabas, P., Gustafsson, J., Anton, M., Cholley, P.E., Huang, S., Gobom, J., Svensson, T., Uhlen, M., Zetterberg, H., Nielsen, J., 2021. Genome-scale metabolic network reconstruction of model animals as a platform for translational research. Proc Natl Acad Sci U S A 118.
